# Mismatch Repair Deficient (dMMR) Colorectal Carcinoma in a Pakistani Cohort: Association With Clinical and Pathological Parameters

**DOI:** 10.7759/cureus.42781

**Published:** 2023-08-01

**Authors:** Atif A Hashmi, Ummara Bukhari, Ramish Rizwan, Faiza Faisal, Ravi Kumar, Umair Arshad Malik, Shamail Zia, Abdur Rahim Khan, Sunder Sham, Muhammad Irfan

**Affiliations:** 1 Pathology, Liaquat National Hospital and Medical College, Karachi, PAK; 2 Internal Medicine, Jinnah Sindh Medical University, Karachi, PAK; 3 General Surgery, Ziauddin University, Karachi, PAK; 4 General Surgery, Dow University of Health Sciences, Karachi, PAK; 5 Internal Medicine, Chandka Medical College, Larkana, PAK; 6 Internal Medicine, Aga Khan University, Karachi, PAK; 7 Pathology, Jinnah Sindh Medical University, Karachi, PAK; 8 Pathology, Karachi Medical and Dental College, Karachi, PAK; 9 Pathology, Lenox Hill Hospital, New York, USA; 10 Statistics, Liaquat National Hospital and Medical College, Karachi, PAK

**Keywords:** colorectal carcinoma, msh2, mlh1, pmmr, dmmr, mismatch repair, msh6, pms2, microsatellite instability, biomarkers

## Abstract

Introduction

Microsatellite instability (MSI) is an important pathway in colorectal carcinoma (CRC) pathogenesis. MSI occurs due to mutations in mismatch repair (MMR) genes that include MutL protein homolog 1 (MLH1), postmeiotic segregation increased 2 (PMS2), MutS homolog 2 (MSH2), and MutS homolog 6 (MSH6). CRC with MSI is termed MMR deficient (dMMR) CRC. Conversely, CRC with intact MMR genes is called microsatellite stable (MSS) or MMR proficient (pMMR). In this study, we compared the clinicopathological features of dMMR CRC with pMMR CRC.

Methods

It was a retrospective study conducted in the Department of Histopathology, Liaquat National Hospital, Karachi, Pakistan, from March 2020 to February 2022, over a duration of two years. Biopsy-proven cases of CRC with upfront surgical resection were included in the study. Microscopic examination was performed to evaluate tumor type, grade, and extent of invasion, presence of necrosis, perineural invasion (PNI), lymphovascular invasion (LVI), peritumoral lymphocytes (PTL), intratumoral lymphocytes (ITL), and nodal metastasis. Immunohistochemical staining was performed using antibodies, namely, MLH1, PMS2, MSH2, and MSH6. Any loss of nuclear expression in tumor cells was termed dMMR or microsatellite instable, whereas the intact nuclear expression in tumor cells was labeled as MSS or pMMR.

Results

A total of 135 cases of CRC were included in the study. The mean age at diagnosis was 46.76 ± 17.74 years, with female predominance (60.7%). The loss of MLH1, PMS2, MSH2, and MSH6 expression was noted in 39.3%, 34.1%, 17.8%, and 16.3% cases, respectively. Overall, 59.3% of CRCs were pMMR, while 40.7% were dMMR. A significant association of MMR status was noted with respect to age, PNI, LVI, tumor grade, tumor (T) and nodal (N) stage, mucinous differentiation, and ITL. dMMR CRC was significantly above 50 years than pMMR CRC. The frequency of PNI and LVI was lower in dMMR CRC than in pMMR CRC. Conversely, the higher grade (grade 3) and higher T-stage (T4) were associated with dMMR CRC. Alternatively, the frequency of higher N stage (N2b) was more commonly seen in pMMR CRC. Moreover, mucinous differentiation and ITL were significantly associated with dMMR CRC.

Conclusion

A significant proportion of CRC patients in our population demonstrated dMMR status. dMMR CRC had a higher histological grade with a higher frequency of mucinous differentiation and higher T-stage. Conversely, the presence of LVI, PNI, and higher N stages were associated with pMMR CRC.

## Introduction

Microsatellites are short tandem repeats of deoxyribonucleic acid (DNA) sequences. Mutations introduced in microsatellites during DNA synthesis are repaired by mismatch repair (MMR) genes. These genes and their coded proteins include MutL protein homolog 1 (MLH1), postmeiotic segregation increased 2 (PMS2), MutS homolog 2 (MSH2), and MutS homolog 6 (MSH6). When defects are present in MMR genes, mutations are introduced during DNA synthesis, resulting in microsatellite instability (MSI) [1]. Many human cancers are associated with MSI [2-4]. MSI is one of the major pathways implicated in colorectal carcinoma (CRC) pathogenesis. CRC with MSI is termed MMR deficient (dMMR) CRC. Conversely, CRC with intact MMR genes is called microsatellite stable (MSS) or MMR proficient (pMMR) [5,6].

Approximately 10-15% of CRCs are dMMR, among which 80% are sporadic owing to MLH1 promoter hypermethylation or v-Raf murine sarcoma viral oncogene homolog B1 (BRAF) mutations, whereas 20% are due to autosomal dominant germline mutations, termed Lynch syndrome (LS) [7]. MSI screening for CRC is initially performed through immunohistochemical (IHC) analysis for MLH1, PMS2, MSH2, and MSH6. The loss of one or two markers mandates MLH1 promoter methylation status and BRAF mutation analysis. The absence of BRAF mutation and MLH1 promoter hypermethylation prompts next-generation sequencing for germline mutations. Previous studies have shown that dMMR CRC is associated with certain histological features, such as mucinous histology, peritumoral lymphocytes (PTL), and intratumoral lymphocytes (ITL) [6]. CRC in the Pakistani population was found to be at a higher tumor (T) and nodal (N) stage than western CRC [8,9]. Clinicopathological features of dMMR CRC have not been widely studied in our population; therefore, we conducted this IHC-based MSI analysis in CRC to better understand the pathogenesis and pathological features of dMMR CRC.

## Materials and methods

It was a retrospective study conducted in the Department of Histopathology, Liaquat National Hospital, Karachi, Pakistan, from March 2020 to February 2022, over a duration of two years. Biopsy-proven cases of CRC with upfront surgical resection were included in the study. Cases with incomplete clinical or pathological records were excluded. Similarly, CRC with neoadjuvant chemotherapy before surgical resection or systemic metastasis at the time of diagnosis was excluded from the analysis. Institutional Review Board (IRB) approval was not needed as it was a retrospective study, and the institution don't mandate IRB approval for retrospective studies.

Surgical specimens were received in the histopathology laboratory, followed by overnight fixation. Gross dimensions of the tumor were recorded, and representative sections were submitted from the tumor along with adjacent normal mucosa and surgical resection margins. Lymph nodes were dissected from the mesenteric tissue and submitted for microscopic examination. Haematoxylin and eosin-stained microscopic sections were examined for tumor type, grade, and extent of invasion, presence of necrosis, perineural invasion (PNI), lymphovascular invasion (LVI), T- and N-stage, PTL, ITL, and nodal metastasis. Histological tumor typing was performed according to the World Health Organization (WHO) classification of the tumors of the digestive tract. Mucinous differentiation was labelled when there were extracellar mucin pools, whereas signet ring differentiation was called when tumor cells show intracytoplasmic mucin vacuole pushing the nucleus to the periphery. Tumors showing more then 50% mucinous and signet ring differentiation were termed mucinous and signet ring adenocarcinoma, respectively. Tumors were graded according to the proportion of gland formation. Well-differentiated/grade 1 tumors had more than 90% gland formation, whereas grade 2 and grade 3 tumors were labeled when tumor showed 6-50% and less than 50% gland formation, respectively. T and N staging was performed according to the American Joint Committee on Cancer (AJCC) staging system. PTL and ITL were categorized into none, mild to moderate, and marked according to the College of American Pathologist's (CAP) guidelines. PTL is labelled when tumor periphery showed lymphoid follicles with germinal center formation. ITL corresponds to lymphocytes within tumor cells. More than three lymphocytes per high-power field were required for marked ITL categorization.

Immunohistochemical analysis

IHC staining was performed using antibodies, namely, MLH1, PMS2, MSH2, and MSH6 antibodies on representative tumor blocks that contained adjacent normal mucosa for the documentation of internal positive control. Any loss of nuclear expression in tumor cells was termed dMMR, whereas intact nuclear expression in all tumor cells was labeled as MSS or pMMR [10].

Statistical analysis

Data analysis was performed using IBM SPSS Statistics for Windows, Version 26.0 (Released 2019; IBM Corp., Armonk, NY). The mean was calculated for patient age, while frequencies and percentages were calculated for other clinicopathological variables. Chi-square and Fisher’s exact tests were applied to determine the association of various clinicopathological features with MMR status. A p-value of <0.05 was considered significant.

## Results

A total of 135 cases of CRC were included in the study. The mean age at diagnosis was 46.76 ± 17.74 years, with female predominance (60.7%). Rectosigmoid was the most common tumor location (67.4%), followed by cecum and splenic flexure (11.9%). The most common tumor type was adenocarcinoma, not otherwise specified (65.2), followed by mucinous carcinoma (24.4%). The most common tumor grade was grade 2 (67.4%), while PNI and LVI were present in 33.3% and 23.7% cases, respectively. Nodal metastasis was seen in 66.7% of cases, with 52.6% demonstrating perinodal extension. Signet ring and mucinous differentiation were present in 18.5% and 36.3% cases, respectively, while marked PTL and ITL were noted in 11.1% and 17.8% cases, respectively. The majority of tumors were at T-stage T3 (71.1%) and N-stage N0 (33.3%). Overall, 59.3% CRCs were pMMR, while, 40.7% were dMMR, as shown in Table 1.

**Table 1:** Clinicopathological features of the population under study SD: standard deviation; NOS: not otherwise specified; T: tumor; N: nodal; MMR: mismatch repair; pMMR: mismatch repair proficient; dMMR: mismatch repair deficient

Clinicopathological features	Values
Age (years)	
Mean ± SD	46.76 ± 17.74
Age groups	
≤50 years, n (%)	72 (53.3)
>50 years, n (%)	63 (46.7)
Gender	
Male, n (%)	53 (39.3)
Female, n (%)	82 (60.7)
Laterality	
Right, n (%)	23 (17)
Left, n (%)	112 (83)
Tumor location	
Cecum, n (%)	16 (11.9)
Ascending colon, n (%)	5 (3.7)
Transverse colon, n (%)	6 (4.4)
Recto-sigmoid, n (%)	91 (67.4)
Descending colon, n (%)	1 (0.7)
Splenic flexure, n (%)	16 (11.9)
Histological features	
Perineural invasion	
Present, n (%)	45 (33.3)
Absent, n (%)	90 (66.7)
Lymphovascular invasion	
Present, n (%)	32 (23.7)
Absent, n (%)	103 (76.3)
Pre-existing polyp	
Present, n (%)	8 (5.9)
Absent, n (%)	127 (94.1)
Tumor type	
Adenocarcinoma, NOS, n (%)	88 (65.2)
Mucinous adenocarcinoma, n (%)	33 (24.4)
Medullary carcinoma, n (%)	9 (6.7)
Signet ring adenocarcinoma, n (%)	5 (3.7)
Tumor differentiation/grade	
Well differentiated/grade 1, n (%)	5 (3.7)
Moderately differentiated/grade 2, n (%)	91 (67.4)
Poorly differentiated/grade 3, n (%)	39 (28.9)
T-stage	
T2, n (%)	11 (8.1)
T3, n (%)	96 (71.1)
T4, n (%)	28 (20.7)
Nodal metastasis	
Present, n (%)	90 (66.7)
Absent, n (%)	45 (33.3)
N-stage	
N0, n (%)	45 (33.3)
N1, n (%)	32 (23.7)
N2a, n (%)	32 (23.7)
N2b, n (%)	26 (19.3)
Perinodal extension	
Present, n (%)	71 (52.6)
Absent, n (%)	64 (47.4)
Signet ring differentiation	
Present, n (%)	25 (18.5)
Absent, n (%)	110 (81.5)
Mucinous differentiation	
Present, n (%)	49 (36.3)
Absent, n (%)	86 (63.7)
Peritumoral lymphocytes	
None, n (%)	104 (77)
Mild to moderate, n (%)	16 (11.9)
Marked, n (%)	15 (11.1)
Intratumoral lymphocytes	
None, n (%)	72 (53.3)
Mild to moderate, n (%)	39 (28.9)
Marked, n (%)	24 (17.8)
Overall MMR status	
pMMR	80 (59.3)
dMMR	55 (40.7)

**Figure 1:**
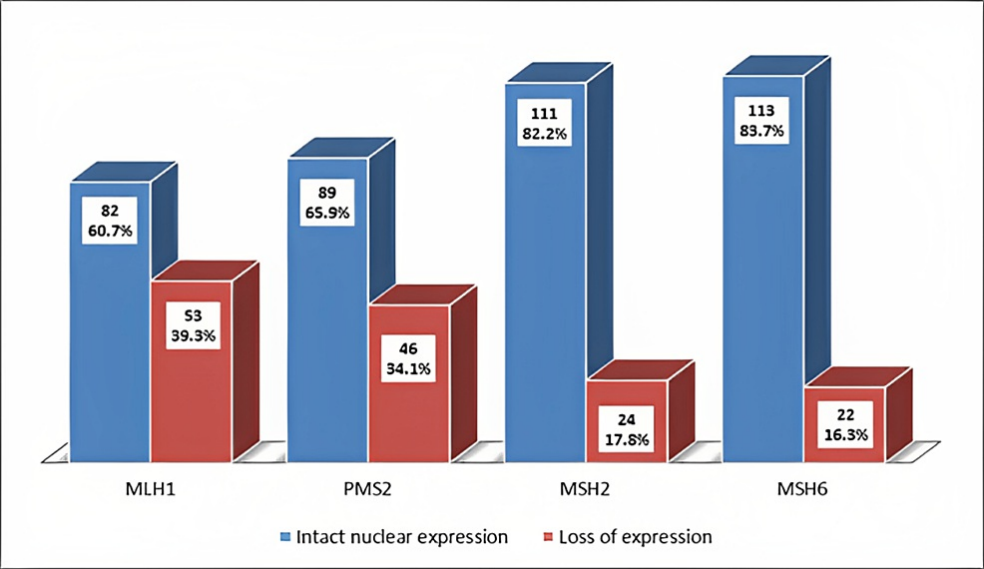
Mismatch repair protein expression by immunohistochemistry MLH1: MutL protein homolog 1; PMS2: postmeiotic segregation increased 2; MSH2: MutS homolog 2; MSH6: MutS homolog 6

The loss of MLH1, PMS2, MSH2, and MSH6 expression was noted in 39.3%, 34.1%, 17.8% and 16.3% cases, respectively, as shown in Figure 1.

Table 2 shows the association of the MMR status of CRC with clinicopathological variables. A significant association of MMR status was noted with respect to age, PNI, LVI, tumor grade, T and N-stage, mucinous differentiation, and ITL. dMMR CRC was significantly above 50 years than pMMR CRC. The frequency of PNI and LVI was lower in dMMR CRC than in pMMR CRC. Conversely, the higher grade (grade 3) and higher T-stage (T4) were associated with dMMR CRC. Alternatively, the frequency of higher N-stage (N2b) was more commonly seen in pMMR CRC. Moreover, mucinous differentiation and ITL were significantly associated with dMMR CRC.

**Table 2:** Comparison of clinicopathological features of colorectal carcinoma with mismatch repair status pMMR: mismatch repair proficient; dMMR: mismatch repair deficient; NOS: not otherwise specified; T: tumor; N: nodal *Chi-square test was applied, **p-value significant as < 0.05, ***Fisher’s exact test was applied.

Clinicopathological features	Values		p-value
	Mismatch repair status		
	pMMR	dMMR	
Gender*			
Male, n (%)	35 (45)	17 (30.9)	0.099
Female, n (%)	44 (55)	38 (69.1)	
Age groups*			
≤50 years, n (%)	52 (65)	20 (36.4)	0.001**
>50 years, n (%)	28 (35)	35 (63.6)	
Laterality*			
Right, n (%)	10 (12.5)	13 (23.6)	0.091
Left, n (%)	70 (87.5)	42 (76.4)	
Location***			
Cecum, n (%)	9 (11.3)	7 (12.7)	0.094
Ascending colon, n (%)	2 (2.5)	3 (5.5)	
Transverse colon, n (%)	3 (3.8)	3 (5.5)	
Recto-sigmoid, n (%)	60 (75)	31 (56.4)	
Descending colon, n (%)	1 (1.3)	0 (0)	
Splenic flexure, n (%)	5 (6.3)	11 (20)	
Histopathological features			
Perineural invasion*			
Present, n (%)	34 (42.5)	11 (20)	0.006**
Absent, n (%)	46 (57.5)	44 (80)	
Lymphovascular invasion*			
Present, n (%)	27 (33.8)	5 (9.1)	0.001**
Absent, n (%)	53 (66.3)	50 (90.9)	
Pre-existing polyp***			
Present, n (%)	3 (3.8)	5 (9.1)	0.270
Absent, n (%)	77 (96.3)	50 (90.9)	
Tumor type***			
Adenocarcinoma, NOS, n (%)	55 (68.8)	33 (60)	0.066
Mucinous adenocarcinoma, n (%)	15 (18.8)	18 (32.7)	
Medullary carcinoma, n (%)	8 (10)	1 (1.8)	
Signet ring cell adenocarcinoma, n (%)	2 (2.5)	3 (5.5)	
Tumor differentiation/grade***			
Well differentiated/grade 1, n (%)	0 (0)	5 (9.1)	0.003**
Moderately differentiated/grade 2, n (%)	61 (76.3)	30 (54.5)	
Poorly differentiated/grade 3, n (%)	19 (23.8)	20 (36.4)	
T-stage***			
T2, n (%)	4 (5)	7 (12.7)	0.002**
T3, n (%)	66 (82.5)	30 (54.5)	
T4, n (%)	10 (12.5)	18 (32.7)	
Nodal metastasis*			
Present, n (%)	56 (70)	34 (61.8)	0.322
Absent, n (%)	24 (30)	21 (38.2)	
N-stage*			
N0, n (%)	24 (30)	21 (38.2)	0.001**
N1, n (%)	21 (26.3)	11 (20)	
N2a, n (%)	12 (15)	20 (36.4)	
N2b, n (%)	23 (28.8)	3 (5.5)	
Perinodal extension*			
Present, n (%)	43 (53.8)	28 (50.9)	0.745
Absent, n (%)	37 (46.3)	27 (49.1)	
Signet ring differentiation*			
Present, n (%)	12 (15)	13 (23.6)	0.204
Absent, n (%)	68 (85)	42 (76.4)	
Mucinous differentiation*			
Present, n (%)	19 (23.8)	30 )54.5)	<0.001**
Absent, n (%)	61 (76.3)	25 (45.5)	
Peritumoral lymphocytes*			
None, n (%)	61 (76.3)	43 (78.2)	0.957
Mild to moderate, n (%)	10 (12.5)	6 (10.9)	
Marked, n (%)	9 (11.3)	6 (10.9)	
Intratumoral lymphocytes*			
None, n (%)	53 (66.3)	19 (34.5)	0.001**
Mild to moderate, n (%)	17 (21.3)	22 (40)	
Marked, n (%)	10 (12.5)	14 (25.5)	

Table 3 shows the association of MLH1 expression with clinicopathological features. A significant association was noted with respect to gender, age, LVI, tumor type, grade, T and N-stage, mucinous differentiation, and ITL. Loss of MLH1 expression was significantly more frequent in females with older age (>50 years). MLH1 loss of expression was also associated with higher grade, mucinous differentiation, and higher T-stage. Moreover, the frequency of LVI in MLH1-deficient tumors was less frequent, whereas marked ITL was more commonly seen in MLH1-deficient tumors.

**Table 3:** Association of MutL protein homolog 1 (MLH1) expression with clinicopathological features MLH1: MutL protein homolog 1; NOS: not otherwise specified; T: tumor; N: nodal *Chi-square test was applied, **p-value significant as < 0.05, ***Fisher’s exact test was applied.

Clinicopathological features	Values		p-value
	MLH1 expression		
	Intact expression/MLH1 procifient	Loss of expression/MLH1 deficient	
Gender*			
Male, n (%)	38 (46.3)	15 (28.3)	0.036**
Female, n (%)	44 (53.7)	38 (71.7)	
Age groups*			
≤50 years, n (%)	51 (62.2)	21 (39.6)	0.010**
>50 years, n (%)	31 (37.8)	32 (60.4)	
Laterality*			
Right, n (%)	12 (14.6)	11 (20.8)	0.356
Left, n (%)	70 (85.4)	42 (79.2)	
Location***			
Cecum, n (%)	11 (13.4)	5 (9.4)	0.228
Ascending colon, n (%)	2 (2.4)	3 (5.7)	
Transverse colon, n (%)	3 (3.7)	3 (5.7)	
Recto-sigmoid, n (%)	59 (72)	32 (60.4)	
Descending colon, n (%)	1 (1.2)	0 (0)	
Splenic flexure, n (%)	6 (7.3)	10 (18.9)	
Histopathological features			
Perineural invasion*			
Present, n (%)	32 (39)	13 (24.5)	0.081
Absent, n (%)	50 (61)	40 (75.5)	
Lymphovascular invasion*			
Present, n (%)	27 (32.9)	5 (9.4)	0.002**
Absent, n (%)	55 (67.1)	48 (90.6)	
Pre-existing polyp***			
Present, n (%)	4 (4.9)	4 (7.5)	0.711
Absent, n (%)	78 (95.1)	49 (92.5)	
Tumor type***			
Adenocarcinoma, NOS, n (%)	58 (70.7)	30 (56.6)	0.001 **
Mucinous adenocarcinoma, n (%)	13 (15.9)	20 (37.7)	
Medullary carcinoma, n (%)	9 (11)	0 (0)	
Signet ring adenocarcinoma, n (%)	2 (2.4)	3 (5.7)	
Tumor differentiation/grade***			
Well differentiated/grade 1, n (%)	0 (0)	5 (9.4)	0.003**
Moderately differentiated/grade 2, n (%)	62 (75.6)	29 (54.7)	
Poorly differentiated/grade 3, n (%)	20 (24.4)	19 (35.8)	
T-stage***			
T2, n (%)	5 (6.1)	6 (11.3)	0.003**
T3, n (%)	67 (81.7)	29 (54.7)	
T4, n (%)	10 (12.2)	18 (34)	
Nodal metastasis*			
Present, n (%)	57 (69.5)	33 (62.3)	0.383
Absent, n (%)	25 (30.5)	20 (37.7)	
N-stage*			
N0, n (%)	25 (30.5)	20 (37.7)	0.001**
N1, n (%)	22 (26.8)	10 (18.9)	
N2a, n (%)	12 (14.6)	20 (37.7)	
N2b, n (%)	23 (28)	3 (5.7)	
Perinodal extension*			
Present, n (%)	43 (52.4)	28 (52.8)	0.965
Absent, n (%)	39 (47.6)	25 (47.2)	
Signet ring differentiation*			
Present, n (%)	12 (14.6)	13 (24.5)	0.148
Absent, n (%)	70 (85.4)	40 (75.5)	
Mucinous differentiation*			
Present, n (%)	18 (22)	31 (58.5)	<0.001**
Absent, n (%)	64 (78)	22 (41.5)	
Peritumoral lymphocytes*			
None, n (%)	61 (74.4)	43 (81.1)	0.547
Mild to moderate, n (%)	10 (12.2)	6 (11.3)	
Marked, n (%)	11 (13.4)	4 (7.5)	
Intratumoral lymphocytes*			
None, n (%)	55 (67.1)	17 (32.1)	<0.001**
Mild to moderate, n (%)	17 (20.7)	22 (41.5)	
Marked, n (%)	10 (12.2)	14 (26.4)	

A significant expression of PMS2 was noted with PNI, LVI, tumor type, grade, T and N-stage, mucinous differentiation, and ITL. The frequency of PNI and LVI in PMS2-deficient tumors was significantly low, whereas PMS2-deficient tumors had higher grade and T-stage. Moreover, the loss of PMS2 was associated with marked ITL and mucinous differentiation, as shown in Table 4.

**Table 4:** Association of postmeiotic segregation increased 2 (PMS2) expression with clinicopathological features PMS2: postmeiotic segregation increased 2; NOS: not otherwise specified; T: tumor; N: nodal *Chi-square test was applied. **Fisher’s exact test was applied. ***p-value significant as < 0.05.

Clinicopathological features	Values		p-value
	PMS2		
	Intact expression/PMS2 proficient	Loss of expression/PMS2 deficient	
Gender*			
Male, n (%)	40 (44.9)	13 (28.3)	0.060
Female, n (%)	49 (55.1)	33 (71.7)	
Age groups*			
≤50 years, n (%)	51 (57.3)	21 (45.7)	0.198
>50 years, n (%)	38 (42.7)	25 (54.3)	
Laterality*			
Right, n (%)	16 (18)	7 (15.2)	0.686
Left, n (%)	73 (82)	39 (84.8)	
Location**			
Cecum, n (%)	13 (14.6)	3 (6.5)	0.086
Ascending colon, n (%)	4 (4.5)	1 (2.2)	
Transverse colon, n (%)	3 (3.4)	3 (6.5)	
Recto-sigmoid, n (%)	62 (69.7)	29 (63)	
Descending colon, n (%)	1 (1.1)	0 (0)	
Splenic flexure, n (%)	6 (6.7)	10 (21.7)	
Histopathological features			
Perineural invasion*			
Present, n (%)	37 (41.6)	8 (17.4)	0.005***
Absent, n (%)	52 (58.4)	38 (82.6)	
Lymphovascular invasion*			
Present, n (%)	32 (36)	0 (0)	<0.001***
Absent, n (%)	57 (64)	46 (100)	
Pre-existing polyp**			
Present, n (%)	6 (6.7)	2 (4.3)	0.716
Absent, n (%)	83 (93.3)	44 (95.7)	
Tumor type**			
Adenocarcinoma, NOS, n (%)	60 (67.4)	28 (60.9)	0.003***
Mucinous adenocarcinoma, n (%)	15 (16.9)	18 (39.1)	
Medullary carcinoma, n (%)	9 (10.1)	0 (0)	
Signet ring adenocarcinoma, n (%)	5 (5.6)	0 (0)	
Tumor differentiation/grade**			
Well differentiated/grade 1, n (%)	0 (0)	5 (10.9)	0.002***
Moderately differentiated/grade 2, n (%)	66 (74.2)	25 (54.3)	
Poorly differentiated/grade 3, n (%)	23 (25.8)	16 (34.8)	
T-stage**			
T2, n (%)	5 (5.6)	6 (13)	0.009***
T3, n (%)	71 (79.8)	25 (54.3)	
T4, n (%)	13 (14.6)	15 (32.6)	
Nodal metastasis*			
Present, n (%)	64 (71.9)	26 (56.5)	0.072
Absent, n (%)	25 (28.1)	20 (43.5)	
N-stage*			
N0, n (%)	25 (28.1)	20 (43.5)	<0.001***
N1, n (%)	22 (24.7)	10 (21.7)	
N2a, n (%)	16 (18)	16 (34.8)	
N2b, n (%)	26 (29.2)	0 (0)	
Perinodal extension*			
Present, n (%)	50 (56.2)	21 (45.7)	0.246
Absent, n (%)	39 (43.8)	25 (54.3)	
Signet ring differentiation*			
Present, n (%)	15 (16.9)	10 (21.7)	0.489
Absent, n (%)	74 (83.1)	36 (78.3)	
Mucinous differentiation*			
Present, n (%)	20 (22.5)	29 (63)	<0.001***
Absent, n (%)	69 (77.5)	17 (37)	
Peritumoral lymphocytes*			
None, n (%)	65 (73)	39 (84.8)	0.275
Mild to moderate, n (%)	13 (14.6)	3 (6.5)	
Marked, n (%)	11 (12.4)	4 (8.7)	
Intratumoral lymphocytes*			
None, n (%)	62 (69.7)	10 (21.7)	<0.001***
Mild to moderate, n (%)	17 (19.1)	22 (47.8)	
Marked, n (%)	10 (11.2)	14 (30.4)	

Table 5 depicts the association of MSH2 expression with clinicopathological parameters. A significant association was noted with LVI, tumor grade, nodal metastasis, T and N stage, perinodal extension, mucinous and signet ring differentiation, and ITL. MSH2-proficient tumors had a higher frequency of LVI, nodal metastasis, perinodal extension, and signet ring differentiation. Conversely, MSH2-deficient tumors were significantly associated with higher grade and T-stage, mucinous differentiation, and ITL. 

**Table 5:** Association of MutS homolog 2 (MSH2) expression with clinicopathological features MSH2: MutS homolog 2; NOS: not otherwise specified; T: tumor; N: nodal *Chi-square test was applied. **Fisher’s exact test was applied. ***p-value significant as < 0.05.

Clinicopathological features	Values		p-value
	MSH2		
	Intact expression/MSH2 proficient	Loss of expression/MSH2 deficient	
Gender*			
Male, n (%)	44 (39.6)	9 (37.5)	0.846
Female, n (%)	67 (60.4)	15 (62.5)	
Age groups*			
≤50 years, n (%)	60 (54.1)	12 (50)	0.718
>50 years, n (%)	51 (45.9)	12 (50)	
Laterality**			
Right, n (%)	18 (16.2)	5 (20.8)	0.559
Left, n (%)	93 (83.8)	19 (79.2)	
Location**			
Cecum, n (%)	14 (12.6)	2 (8.3)	0.300
Ascending colon, n (%)	5 (4.5)	0 (0)	
Transverse colon, n (%)	3 (2.7)	3 (12.5)	
Recto-sigmoid, n (%)	76 (68.5)	15 (62.5)	
Descending colon, n (%)	1 (0.9)	0 (0)	
Splenic flexure, n (%)	12 (10.8)	4 (16.7)	
Histopathological features			
Perineural invasion*			
Present, n (%)	40 (36)	5 (20.8)	0.152
Absent, n (%)	71 (64)	19 (79.2)	
Lymphovascular invasion*			
Present, n (%)	32 (28.8)	0 (0)	0.003***
Absent, n (%)	79 (71.2)	24 (100)	
Pre-existing polyp**			
Present, n (%)	7 (6.3)	1 (4.2)	1.000
Absent, n (%)	104 (93.7)	23 (95.8)	
Tumor type**			
Adenocarcinoma, NOS, n (%)	76 (68.5)	12 (50)	0.065
Mucinous adenocarcinoma, n (%)	22 (19.8)	11 (45.8)	
Medullary carcinoma, n (%)	8 (7.2)	1 (4.2)	
Signet ring adenocarcinoma, n (%)	5 (4.5)	0 (0)	
Tumor differentiation/grade**			
Well differentiated/grade 1, n (%)	2 (1.8)	3 (12.5)	0.011***
Moderately differentiated/grade 2, n (%)	80 (72.1)	11 (45.8)	
Poorly differentiated/grade 3, n (%)	29 (26.1)	10 (41.7)	
T-stage**			
T2, n (%)	7 (6.3)	4 (16.7)	<0.001***
T3, n (%)	88 (79.3)	8 (33.3)	
T4, n (%)	16 (14.4)	12 (50)	
Nodal metastasis*			
Present, n (%)	83 (74.8)	7 (29.2)	<0.001***
Absent, n (%)	28 (25.2)	17 (70.8)	
N-stage*			
N0, n (%)	28 (25.2)	17 (70.8)	<0.001***
N1, n (%)	28 (25.2)	4 (16.7)	
N2a, n (%)	29 (26.1)	3 (12.5)	
N2b, n (%)	26 (23.4)	0 (0)	
Perinodal extension*			
Present, n (%)	68 (61.3)	3 (12.5)	<0.001***
Absent, n (%)	43 (38.7)	21 (87.5)	
Signet ring differentiation**			
Present, n (%)	25 (22.5)	0 (0)	0.007***
Absent, n (%)	86 (77.5)	24 (100)	
Mucinous differentiation*			
Present, n (%)	31 (27.9)	18 (75)	<0.001**
Absent, n (%)	80 (72.1)	6 (25)	
Peritumoral lymphocytes**			
None, n (%)	85 (76.6)	19 (79.2)	1.000
Mild to moderate, n (%)	13 (11.7)	3 (12.5)	
Marked, n (%)	13 (11.7)	2 (8.3)	
Intratumoral lymphocytes**			
None, n (%)	70 (63.1)	2 (8.3)	<0.001***
Mild to moderate, n (%)	27 (24.3)	12 (50)	
Marked, n (%)	14 (12.6)	10 (41.7)	

Table 6 presents the association of MSH6 expression with clinical and pathological features. A significant association of MSH 6 expression was noted with PNI, LVI, nodal metastasis, tumor grade, T and N-stage, perinodal extension, signet ring and mucinous differentiation, and ITL. MSH6-proficient tumors had a higher frequency of PNI, LVI, signet ring differentiation, nodal metastasis, and perinodal extension. Alternatively, MSH6-deficient tumors had higher grade and T-stage, higher frequency of mucinous differentiation, and marked ITL.

**Table 6:** Association of MutS homolog 6 (MSH6) expression with clinicopathological features MSH6: MutS homolog 6; NOS: not otherwise specified; T: tumor; N: nodal

Clinicopathological features	Values		p-value
	MSH6		
	Intact expression/MSH6 proficient	Loss of expression/MSH6 deficient	
Gender*			
Male, n (%)	46 (40.7)	7 (31.8)	0.435
Female, n (%)	67 (59.3)	15 (68.2)	
Age groups*			
≤50 years, n (%)	62 (54.9)	10 (45.5)	0.418
>50 years, n (%)	51 (45.1)	12 (54.5)	
Laterality**			
Right, n (%)	18 (15.9)	5 (22.7)	0.534
Left, n (%)	95 (84.1)	17 (77.3)	
Location**			
Cecum, n (%)	14 (12.4)	2 (9.1)	0.214
Ascending colon, n (%)	5 (4.4)	0 (0)	
Transverse colon, n (%)	3 (2.7)	3 (13.6)	
Recto-sigmoid, n (%)	78 (69)	13 (59.1)	
Descending colon, n (%)	1 (0.9)	0 (0)	
Splenic flexure, n (%)	12 (10.6)	4 (18.2)	
Histopathological features			
Perineural invasion*			
Present, n (%)	42 (37.2)	3 (13.6)	0.032***
Absent, n (%)	71 (62.8)	19 (86.4)	
Lymphovascular invasion*			
Present, n (%)	32 (28.3)	0 (0)	0.004***
Absent, n (%)	81 (71.7)	22 (100)	
Pre-existing polyp**			
Present, n (%)	7 (6.2)	1 (4.5)	1.000
Absent, n (%)	106 (93.8)	21 (95.5)	
Tumor type**			
Adenocarcinoma, NOS, n (%)	76 (67.3)	12 (54.5)	0.251
Mucinous adenocarcinoma, n (%)	24 (21.2)	9 (40.9)	
Medullary carcinoma, n (%)	8 (7.1)	1 (4.5)	
Signet ring adenocarcinoma, n (%)	5 (4.4)	0 (0)	
Tumor differentiation/grade**			
Well differentiated/grade 1, n (%)	2 (1.8)	3 (13.6)	0.003***
Moderately differentiated/grade 2, n (%)	82 (72.6)	9 (40.9)	
Poorly differentiated/grade 3, n (%)	29 (25.7)	10 (45.5)	
T-stage**			
T2, n (%)	7 (6.2)	4 (18.2)	<0.001***
T3, n (%)	90 (79.6)	6 (27.3)	
T4, n (%)	16 (14.2)	12 (54.5)	
Nodal metastasis*			
Present, n (%)	83 (73.5)	7 (31.8)	<0.001***
Absent, n (%)	30 (26.5)	15 (68.2)	
N-stage*			
N0, n (%)	30 (26.5)	15 (68.2)	0.001***
N1, n (%)	28 (24.8)	4 (18.2)	
N2a, n (%)	29 (25.7)	3 (13.6)	
N2b, n (%)	26 (23)	0 (0)	
Perinodal extension*			
Present, n (%)	68 (60.2)	3 (13.6)	<0.001***
Absent, n (%)	45 (39.8)	19 (86.4)	
Signet ring differentiation**			
Present, n (%)	25 (22.1)	0 (0)	0.013***
Absent, n (%)	88 (77.9)	22 (100)	
Mucinous differentiation*			
Present, n (%)	33 (29.2)	16 (72.7)	<0.001***
Absent, n (%)	80 (70.8)	6 (27.3)	
Peritumoral lymphocytes**			
None, n (%)	87 (77)	17 (77.3)	0.922
Mild to moderate, n (%)	13 (11.5)	3 (13.6)	
Marked, n (%)	13 (11.5)	2 (9.1)	
Intratumoral lymphocytes**			
None, n (%)	70 (61.9)	2 (9.1)	<0.001**
Mild to moderate, n (%)	27 (23.9)	12 (54.5)	
Marked, n (%)	16 (14.2)	8 (36.4)	

## Discussion

We found a relatively high dMMR CRC in our population (40.7%). MLH1 was the most frequently deficient MMR marker in our cohort of cases, followed by PMS2, MSH2, and MSH6. dMMR CRCs were associated with mucinous histology and ITL. With respect to prognostic parameters, dMMR CRCs were associated with higher grade and T-stage, whereas the frequency of PNI, LVI, and higher N-stage was lower.

There has been an interest in dMMR CRC recently owing to differences in prognosis and the role of immunotherapy. dMMR CRC can be due to germline mutations owing to LS or sporadic BRAF mutations or MLH1 promoter hypermethylation. Errors introduced during DNA replication are corrected by the function of two pairs of genes: MLH1 and PMS; and MSH2 and MSH6. MLH is needed for the proper function of PMS2, whereas MSH2 is required for MSH6 functioning. IHC analysis for MLH1, PMS2, MSH2, and MSH6 is initially indicated and specific staining combinations predict sporadic vs. familial dMMR CRC [1,5].

The frequency of dMMR CRC varies in different studies. A Chinese study involving 133 cases of CRC revealed a loss of expression of MLH1, MSH2, MSH6, and PMS2 in 55.6%, 33.8%, 41.4%, and 57.9% cases, respectively. They found a significant association of dMMR status with age, gender, and tumor site, whereas no significant association was noted with T- and N-stages [11]. Conversely, Liang et al. [12] in a study including 61 CRC patients concluded that there was a significant association of dMMR status with age (<55 years), female gender, location (right colon), tumor size (>5 cm), T-stage (T4), high grade, and mucinous differentiation. We also found a significant association of dMMR CRC with T4 stage, higher tumor grade, and mucinous differentiation, however, the association of dMMR CRC with younger age was not established in our study. Similarly, Liang et al. [12] did not find any association of dMMR status with LVI, as noted in our study. Sacdalan et al. [13] also reported aggressive histological features (poor differentiation, mucinous histology) of dMMR CRC in Filipino patients. Concordant with our findings, mucinous and poor differentiation were found in dMMR CRC [14].

Other histological features of dMMR CRC include the presence of immune cell infiltration (PTL and ITL). PTL corresponds to a Crohn’s-like inflammatory reaction at the periphery of the tumor, whereas ITL is the presence of inflammatory cells within the cancer cells. This unique feature of dMMR CRC was attributed to elevated mutational burden and neoantigen overload that results in an immune reaction against tumor cells [15]. We found a significant association of dMMR CRC with ITL, whereas no significant association was established with PTL.

Despite aggressive histological features, there is a survival benefit in CRC with dMMR status. Previous studies have reported better overall survival in dMMR CRC than pMMR CRC, especially in early-stage CRC [16]. We did not evaluate survival in our study but found a significant association of dMMR status with lack of PNI, LVI, and lower N-stage signifying prognostically better pathological features in dMMR CRC.

Another significance of dMMR status is the predictive role of immunotherapy. There is a promising role of immunotherapy in dMMR metastatic CRC, while its role in pMMR CRC is being sought [17]. We did not evaluate the predictive value of dMMR status in our study. 

Limitations

The main limitation of our study was the lack of clinical follow-up to evaluate the overall and disease-free survival of dMMR CRC. Second, molecular studies for BRAF, MLH1 promoter hypermethylation, and next-generation sequencing for germline mutations for LS were not performed. Additionally, there is an increasing interest in the role of immunotherapy in dMMR CRC that we did not evaluate in our study. Moreover, this was a single-center study; therefore, the sample size was also limited.

## Conclusions

In this study, we evaluated dMMR status in CRC and found that a significant proportion of CRC cases were dMMR in our study population. We noted that poor differentiation (high grade) and mucinous histology were associated with dMMR CRC. Moreover, dMMR CRC was of higher T-stage than pMMR in our study. Conversely, dMMR CRC was associated with better prognostic features, such as lower N-stage and lower frequency of PNI and LVI. Due to these distinctive features of dMMR CRC, evaluating the dMMR status in CRC is of utmost importance. Our study was limited because we did not evaluate the predictive value of dMMR status in CRC, and the frequency of germline mutations with dMMR CRC was not sought in our study.

## References

[1] Olave MC, Graham RP (2022). Mismatch repair deficiency: the what, how and why it is important. Genes Chromosomes Cancer.

[2] Hause RJ, Pritchard CC, Shendure J, Salipante SJ (2016). Classification and characterization of microsatellite instability across 18 cancer types. Nat Med.

[3] Hashmi AA, Mudassir G, Hashmi RN (2019). Microsatellite instability in endometrial carcinoma by immunohistochemistry, association with clinical and histopathologic parameters. Asian Pac J Cancer Prev.

[4] Puliga E, Corso S, Pietrantonio F, Giordano S (2021). Microsatellite instability in gastric cancer: between lights and shadows. Cancer Treat Rev.

[5] Vilar E, Gruber SB (2010). Microsatellite instability in colorectal cancer-the stable evidence. Nat Rev Clin Oncol.

[6] Hashmi AA, Ali R, Hussain ZF (2017). Mismatch repair deficiency screening in colorectal carcinoma by a four-antibody immunohistochemical panel in Pakistani population and its correlation with histopathological parameters. World J Surg Oncol.

[7] Sinicrope FA, Sargent DJ (2012). Molecular pathways: microsatellite instability in colorectal cancer: prognostic, predictive, and therapeutic implications. Clin Cancer Res.

[8] Hashmi AA, Hashmi SK, Ali N (2018). Clinicopathologic features of colorectal carcinoma: features predicting higher T-stage and nodal metastasis. BMC Res Notes.

[9] Hashmi AA, Aslam M, Rashid K (2023). Early-onset/young-onset colorectal carcinoma: a comparative analysis of morphological features and biomarker profile. Cureus.

[10] Markow M, Chen W, Frankel WL (2017). Immunohistochemical pitfalls: common mistakes in the evaluation of lynch syndrome. Surg Pathol Clin.

[11] Li J, Xu Q, Luo C, Chen L, Ying J (2020). Clinicopathologic characteristics of resectable colorectal cancer with mismatch repair protein defects in Chinese population: retrospective case series and literature review. Medicine (Baltimore).

[12] Liang Y, Cai X, Zheng X, Yin H (2021). Analysis of the clinicopathological characteristics of stage I-III colorectal cancer patients deficient in mismatch repair proteins. Onco Targets Ther.

[13] Sacdalan DL, Garcia RL, Diwa MH, Sacdalan DB (2021). Clinicopathologic factors associated with mismatch repair status among Filipino patients with young-onset colorectal cancer. Cancer Manag Res.

[14] Chen J, Zhou L, Gao J, Lu T, Wang J, Wu H, Liang Z (2020). Clinicopathological characteristics and mutation spectrum of colorectal adenocarcinoma with mucinous component in a Chinese cohort: comparison with classical adenocarcinoma. Front Oncol.

[15] Ooki A, Shinozaki E, Yamaguchi K (2021). Immunotherapy in colorectal cancer: current and future strategies. J Anus Rectum Colon.

[16] Jin Z, Sinicrope FA (2021). Prognostic and predictive values of mismatch repair deficiency in non-metastatic colorectal cancer. Cancers (Basel).

[17] Ganesh K, Stadler ZK, Cercek A, Mendelsohn RB, Shia J, Segal NH, Diaz LA Jr (2019). Immunotherapy in colorectal cancer: rationale, challenges and potential. Nat Rev Gastroenterol Hepatol.

